# ECoG Beta Suppression and Modulation During Finger Extension and Flexion

**DOI:** 10.3389/fnins.2020.00035

**Published:** 2020-02-13

**Authors:** Julian Unterweger, Martin Seeber, Stavros Zanos, Jeffrey G. Ojemann, Reinhold Scherer

**Affiliations:** ^1^Institute of Neural Engineering, Graz University of Technology, Graz, Austria; ^2^Functional Brain Mapping Laboratory, Department of Fundamental Neurosciences, University of Geneva, Geneva, Switzerland; ^3^Translational Neurophysiology Laboratory, Institute of Bioelectronic Medicine, Feinstein Institutes for Medical Research, Manhasset, NY, United States; ^4^Department of Neurological Surgery, University of Washington, Seattle, WA, United States; ^5^Brain-Computer Interfaces and Neural Engineering Laboratory, School of Computer Science and Electronic Engineering, University of Essex, Colchester, United Kingdom

**Keywords:** electrocorticogram, brain-computer interface, beta band, high gamma, movement-phase related amplitude modulation

## Abstract

Neural oscillations originate predominantly from interacting cortical neurons and consequently reflect aspects of cortical information processing. However, their functional role is not yet fully understood and their interpretation is debatable. Amplitude modulations (AMs) in alpha (8–12 Hz), beta (13–30 Hz), and high gamma (70–150 Hz) band in invasive electrocorticogram (ECoG) and non-invasive electroencephalogram (EEG) signals change with behavior. Alpha and beta band AMs are typically suppressed (desynchronized) during motor behavior, while high gamma AMs highly correlate with the behavior. These two phenomena are successfully used for functional brain mapping and brain-computer interface (BCI) applications. Recent research found movement-phase related AMs (MPA) also in high beta/low gamma (24–40 Hz) EEG rhythms. These MPAs were found by separating the suppressed AMs into sustained and dynamic components. Sustained AM components are those with frequencies that are lower than the motor behavior. Dynamic components those with frequencies higher than the behavior. In this paper, we study ECoG beta/low gamma band (12–30 Hz/30–42 Hz) AM during repetitive finger movements addressing the question whether or not MPAs can be found in ECoG beta band. Indeed, MPA in the 12–18 Hz and 18–24 Hz band were found. This additional information may lead to further improvements in ECoG-based prediction and reconstruction of motor behavior by combining high gamma AM and beta band MPA.

## 1. Introduction

**Figure 1:**
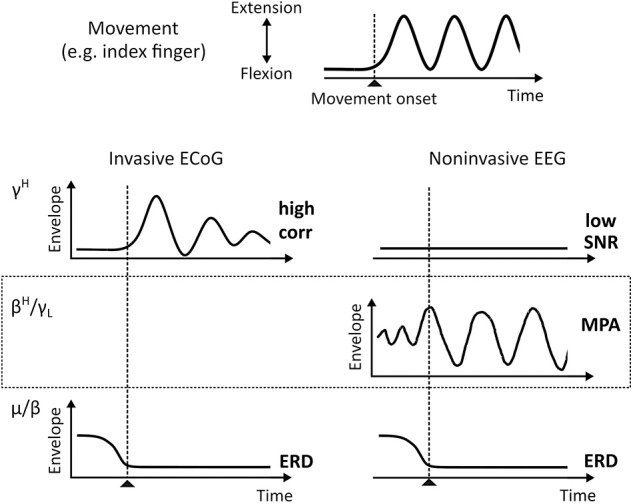
ECoG and EEG envelopes of known oscillatory phenomena during single-trial motor behavior (idealized). The top plot shows a typical times series recorded from data glove sensors during, for example, index finger extension and flexion movements. Below characteristic γ^*H*^ (70–150 Hz) and μ/β (8–12 Hz/13–30 Hz) activities for invasive ECoG and noninvasive EEG, respectively, are shown. EEG β_*H*_-γ_*L*_ (24–40 Hz) MPA is shown in the dotted box. *corr*, Pearson Correlation Coefficient; ERD, Event-Related Desynchronization; MPA, Movement Phase related Amplitude modulation.

Functional brain mapping (fBM) and brain-computer interface (BCI) technologies identify behavior—cognitive and motor—by interpretation of brain signal patterns. For example, invasive electrocorticogram (ECoG) high gamma band (70–150 Hz) activity (γ^*H*^) strongly correlates with motor behavior (Crone et al., 1998; Edwards et al., 2005; Miller et al., 2007, 2014; Schalk et al., 2007; Scherer et al., 2009; Martin et al., 2016) and was suggested to contain similar information as firing rates on a intermediate spatial scale (Ray et al., 2008; Manning et al., 2009; Miller et al., 2009b). The single-trial signal-to-noise ratio (SNR) of γ^*H*^ is high, which is essential for robust and timely online BCI performance. γ^*H*^ can also be found in the noninvasive electroencephalogram (EEG) (Ball et al., 2008; Darvas et al., 2010; Grosse-Wentrup et al., 2011; Seeber et al., 2015); However, the single-trial SNR is low in non-invasive EEG. In contrast, oscillations over sensorimotor areas in the μ (8–12 Hz) and β (13–30 Hz) frequency range are much more pronounced in EEG recordings on a single-trial level. The suppression of theses rhythms—a phenomenon known as event-related desynchronization (ERD) (Pfurtscheller and Da Silva, 1999)—were suggested to represent increased excitability in underlying neural circuitry (Neuper and Pfurtscheller, 2001) or a release of inhibition facilitating movement initiation (Hermes et al., 2012). Sensorimotor μ and β band suppression during motor behavior is also characteristic for ECoG. A simplified, idealized representation of event-related μ, β, and γ^*H*^ activity patterns during movement are summarized in Figure 1. Since these patterns are well described in the literature, they are commonly used in BCI. Currently a precise reconstruction of the behavior from these macroscopic recordings is, however, only possible to a limited extent. To improve fBM/BCI performance, it is essential to deepen our understanding of signals recorded as local field potentials (LFP), ECoG, and EEG.

Recently, we started to systematically study EEG source dynamics during upright gait (Wagner et al., 2012; Seeber et al., 2014). Results confirmed a sustained μ and β band ERD and γ^*H*^ activity during walking when compared to standing. Additionally, we found EEG source amplitudes in the high β-low γ (β^*H*^-γ_*L*_) frequency range (24–40 Hz) that are modulated in relation to the gait cycle (Wagner et al., 2012, 2016; Seeber et al., 2014, 2015). These movement-phase related amplitude modulations (MPA, see Figure 1) showed different spectral profiles than classical ERD and event-related synchronization (ERS) phenomena (Pfurtscheller and Da Silva, 1999; Neuper and Pfurtscheller, 2001). We found β^*H*^-γ_*L*_ MPA being present during rhythmic finger extension and flexion movements using EEG source reconsturction (Seeber et al., 2016). Because spectral profiles were suggested to be characteristic for specific large-scale networks (Donner and Siegel, 2011; Siegel et al., 2012), we interpreted MPA to represent different frequency-specific networks than classical ERD/ERS (Seeber et al., 2014, 2016). Moreover, following previous literature (Neuper and Pfurtscheller, 2001; Hermes et al., 2012) sustained ERD/ERS phenomena, i.e., different synchrony states in sensorimotor populations, during repetitive movements indicate the contrast between non-movement and active movement states. The functional meaning of MPA is less clear so far. Based on their time-frequency properties and cortical location we suggested that they might reflect processes linked to the prediction and integration of sensorimotor information (Seeber et al., 2016). Yet, more work is needed to falsify or support this viewpoint.

Since μ, β, and γ^*H*^ are phenomena found during repetitive finger extension and flexion movements in both ECoG and EEG, in this work, we investigate whether MPA in β_*H*_-γ_*L*_ range can also be found in ECoG. This would complement the gap in Figure 1. As outlined above, our hypothesis is that β_*H*_-γ_*L*_ envelopes, i.e., band-pass filtered power signals tat are commonly used for movement decoding, are composed by superposition of functionally different frequency-specific cortical networks. The first class of networks provides information on the movement state (motor system “active” or “inactive”). These networks contribute elements of sustained amplitude modulation during repetitive movements and are linked to classical ERD/ERS. The second class of networks provide information on functional aspects of the motor behavior (movement phases). These networks contribute elements of dynamic amplitude modulations and are linked to MPAs. Note that interpretation of MPAs is only meaningful when the motor system is “active.” To test this perspective, we split up β and γ envelopes in sustained and dynamic components, and compare their correlation with behavior, precisely movement trajectories recorded with a data glove. Sustained and dynamic components can be decomposed by low and high pass filters, respectively. The movement pace defines the filter cut-off frequency. Modulation frequencies close to, but below the movement pace reflect ERD/ERS. Modulation frequencies close to, but above the movement pace might show MPAs.

## 2. Methods

### 2.1. Patients, Data Acquisition, and Experimental Paradigm

The study participants were six neurosurgical patients with intractable epilepsy (Patient ID: BP, CC, MN, OJ, ES, and DJ). They underwent temporary placement of a subdural electrode array (8 × 8 grid, 1 cm horizontal and vertical inter-electrode distance) to localize the epileptic seizure focus and map brain function prior to surgical resection. Electrode placement was determined by clinical considerations, with the necessity and location of the electrodes determined by the interdisciplinary conference of the Regional Epilepsy Center, Harborview Medical Center, University of Washington. The patients gave informed consent prior to participation in a manner approved by the Human Studies Division (Institutional Review Board) of the University of Washington.

ECoG signals were recorded on a Synamp2 amplifier (Compumedics Neuroscan) at a sampling rate of 2,000 samples per second (1,000 for patients BP and CC) and band-pass filtered between 1 and 500 (200 for patients BP and CC) Hz. The position of each finger was registered through a 5-degrees of freedom data glove device (Fifth Dimension Technologies, Inc.) with a rate of 25 samples per second.

Participants were asked to perform a cue-guided repetitive motion task of individual finger movements. Two-second-long visual cues for thumb, index finger and a pinching motion (involving thumb and index finger movement as well as middle finger, ring finger and pinky) were randomly interleaved and separated by 2-s rest intervals. The cues were delivered visually on a 10 by 10-cm presentation window at a distance of 70 cm from the subject, using the BCI2000 software (Schalk et al., 2004). In total there were 29–31 cue presentations per type of visual cue (except for one subject which was only presented with 23–26 cues per type of visual cue). The results in this paper focus on thumb and index finger movements only.

### 2.2. Data Analysis

ECoG time series were down-sampled to 1,000 samples per second and visually inspected for the presence of artifacts. Noisy segments and malfunctioning channels were removed. Overall, 92.6% of channels and 91.5% of movement trials were retained for further analysis. The down-sampled ECoG data was band pass filtered between 0.1 and 200 Hz (8*th* order Butterworth IIR filter) and re-referenced with respect to the common average. Data glove recordings were up-sampled by zero-order-hold interpolation to 1000 samples per second. Thumb and index finger movement onset and movement duration were selected by visual inspection.

The β-γ_*L*_ frequency range was subdivided into five non-overlapping sub-bands β_1_ = 12 − 18*Hz*, β_2_ = 18 − 24*Hz*, β_3_ = 24 − 30*Hz*, γ_1_ = 30 − 36*Hz*, γ_2_ = 36 − 42*Hz*. The Hilbert transform was applied to the β_*i*_ and γ_*i*_ band pass filtered re-referenced ECoG signals (6^*th*^ order Butterworth IIR filter) to compute the time varying analytical amplitude ${\hat{A}}_{j}$ (*j* = [*thumb, index*]), which is a measure of amplitude modulation (AM). Additionally, the analytical amplitude ${\hat{A}}_{j}$ in the high γ^*H*^ = 70 − 150*Hz* frequency band was computed. This resulted in six (frequency band $fb=\left[\beta_{1},\beta_{2},\beta_{3},\gamma_{1},\gamma_{2},\gamma^{H}\right]$) analytical amplitude *Â*_*j,ch,fb*_ time series per channel *ch* = 1, 2, …64.

**Figure 2:**
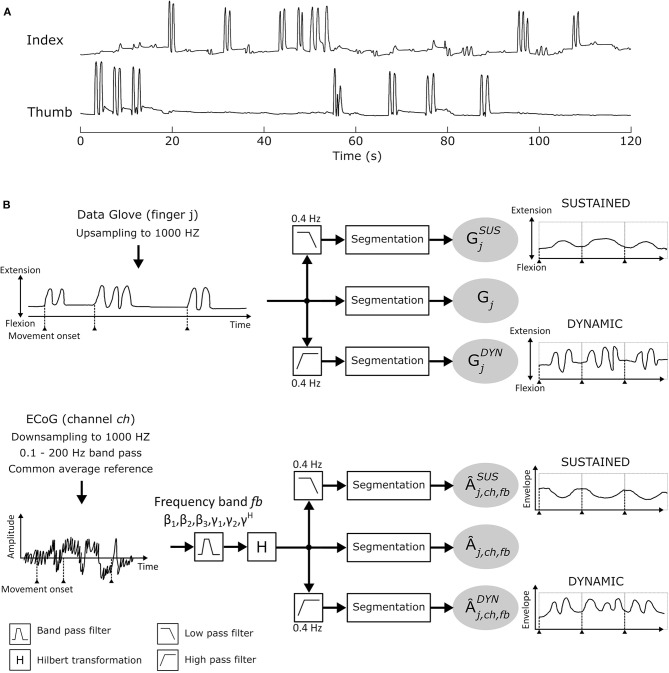
**(A)** Individual thumb and index finger trajectories recorded with the 5-DOF data-glove for one patient in a time window of 120 s. **(B)** Signal analysis pipeline.

Study participants performed between 1 and 6 finger movement cycles per trial (see Figure 2A for finger movement trajectories). This corresponds to movement frequencies from 0.5 to 3 Hz. In order to sufficiently separate sustained (condition SUS) and dynamic (condition DYN) components, a cut-off frequency of 0.4 Hz was selected. Hence, each *Â*_*j,ch,fb*_ was further divided into sustained ${\hat{A}}_{j,ch,fb}^{SUS}$ and dynamic ${\hat{A}}_{j,ch,fb}^{DYN}$ AM components by applying a 0.4*Hz* low pass and high pass filter, respectively. Data glove time series *G*_*j*_ (*j* = [*thumb, index*]) was also subdivided into sustained $G_{j}^{SUS}$ (≤ 0.4 Hz) and dynamic $G_{j}^{DYN}$ (>0.4 Hz) elements. A 6*th* order Butterworth IIR low (high) pass filter was used. From each of the calculated time series, 4 s segments were extracted from [− 1.0 … 3.0]*s* with respect to movement onset *t* = 0 and concatenated. Further analyses were made with these new time series. Figure 2B summarizes the signal processing pipeline.

Pearson correlation coefficients $corr\left(G_{j}^{SUS},{\hat{A}}_{j,ch,fb}^{SUS}\right)$, and $corr\left(G_{j}^{DYN},{\hat{A}}_{j,ch,fb}^{DYN}\right)$ were computed for each finger *j*, frequency band *fb* and channel *ch*. As reference, gold standard correlations *corr*(*G*_*j*_, *Â*_*j,ch,fb*_) were calculated, without separating sustained and dynamic AM.

The entire correlation-analysis was repeated with *N* = 1, 000 random time-domain permutations of common-average re-referenced channel data. The obtained correlation values where then pooled and permutation distribution for the different conditions and frequency bands was evaluated. Permutation distribution showed to be normal for all patients for each frequency band and condition. Nonetheless sub-band standard deviation showed to be higher compared to γ^*H*^ and random permutations of *corr*(*G*_*j*_, *Â*_*j,ch,fb*_) exhibited the lowest and $corr\left(G_{j}^{SUS},{\hat{A}}_{j,ch,fb}^{SUS}\right)$ the highest standard deviations over-all. To gain comparability between frequency bands and conditions, Pearson-correlation coefficients were converted into z-scores *z*_*j,ch,fb*_, $z_{j,ch,fb}^{SUS}$ and $z_{j,ch,fb}^{DYN}$ by subtracting the mean and dividing by the standard deviation of the underlying pooled permutation distribution. Z-scores give the distance from the mean and are measured in standard deviations. The 2.5% and 97.5% quantile were selected as subject-specific chance level for negative and positive z-scores, respectively, conforming with two times the standard deviation, hence a z-score of approximately two.

Z-scores that exceed chance level show a significant relation between ECoG AM envelopes and finger movement trajectories. We defined these AM envelopes as MPAs. For visual presentation envelopes of the channel with the highest z-score located over movement-related areas were averaged after trial-wise segmentation for each patient and frequency band.

## 3. Results

**Figure 3:**
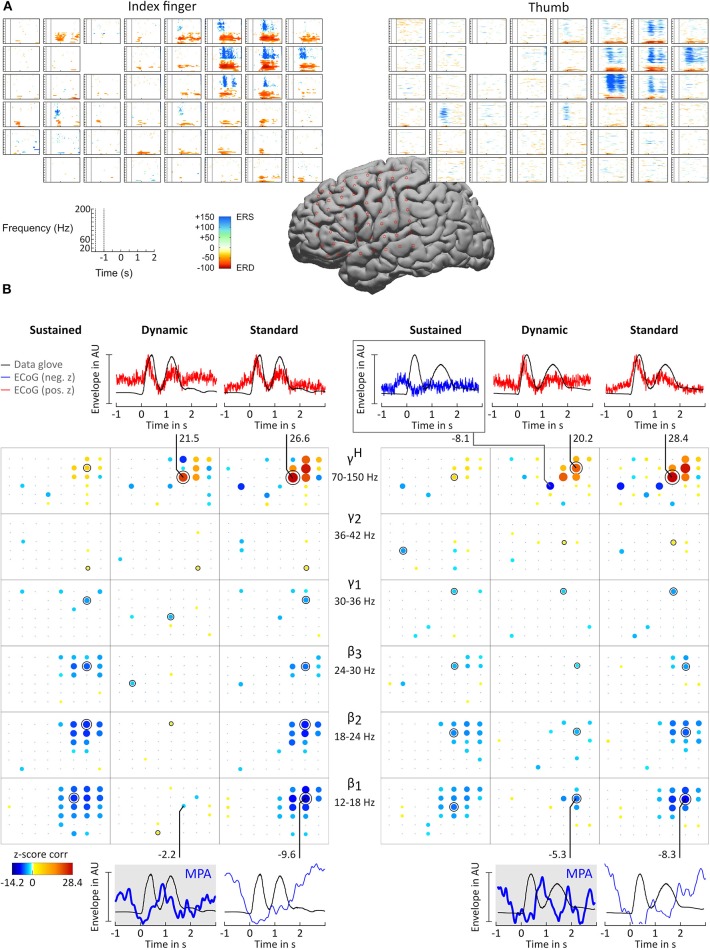
Results for subject BP. **(A)** ERD/ERS time-frequency maps. The plots show, topographically arranged (8 × 8 grid), significant ERD and ERS activity plots for index finger (left) and thumb (right). Electrode locations are marked by star symbol on standard brain. **(B)** Correlation analysis and MPA. Significant z-score transformed Pearson correlation coefficients, computed between corresponding digit trajectory and ECoG envelope components, are displayed for index finger (left) and thumb (right) movements. Z-scores are topographically arranged for each condition (columns, sustained, dynamic, and standard) and frequency band (rows, β_1_ = 12 − 18 Hz, β_2_ = 18 − 24 Hz, β_3_ = 24 − 30 Hz, γ_1_ = 30 − 36 Hz, γ_2_ = 36 − 42 Hz, and γ^*H*^ = 70 − 150 Hz) independently. Size and color of bubbles correspond to z-score values. A black “x” symbol marks channels with z-scores below chance level. A black annulus marks channels with the highest absolute value for each frequency band. Blank spaces in the 8 × 8 electrode grid mark channels excluded from the analysis. Note that negative correlations were smaller than positive correlations. To enhance readability of the bubble plots negative correlations are doubled in size. For selected sensorimotor channels curves of averaged amplitude envelopes of filtered ECoG and averaged data-glove trajectory for β_1_ (bottom) and γ^*H*^ (top) frequency bands are plotted. The number next to the line connecting channels and plots are the corresponding z-scores. β_1_ MPAs are drawn with thicker lines and highlighted in gray background color.

ERD/ERS time-frequency maps (Graimann et al., 2002) were computed for each patient to obtain a reference image of β and γ^*H*^ activity. ERD/ERS maps are time-frequency plots that display significant ERD and ERS in predefined frequency bands. Topographically arranged, they give a clear overview of the movement-related behavior of the non-phase locked activity over a broad frequency range. Figure 3A shows example ERD/ERS maps for patient BP index finger and thumb movement. The maps show widespread β band ERD during finger flexion and extension over sensorimotor areas and more focal high γ activity over cortical index finger and thumb representation areas. This pattern was visible in all patients. For patient ES the pattern was widespread and distributed over the whole grid.

**Figure 4:**
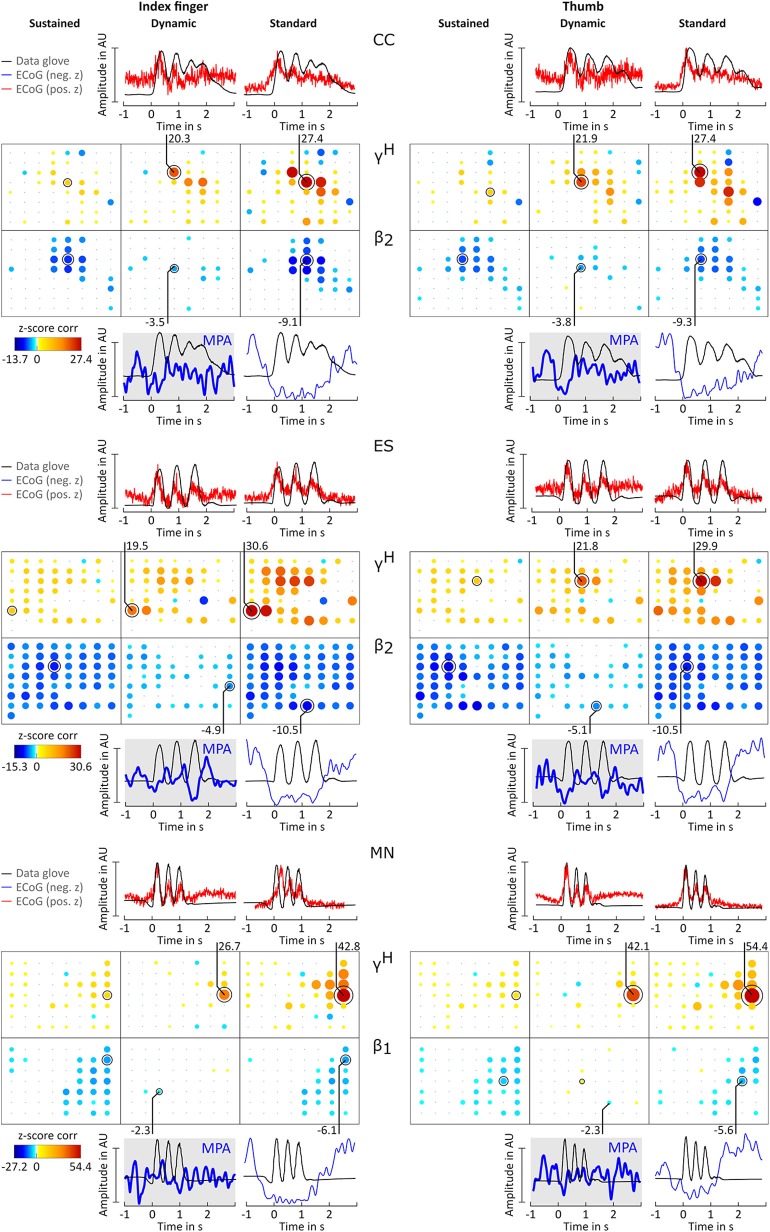
Correlation analysis results and MPA for subjects CC, ES, and MN. Significant z-score transformed Pearson correlation coefficients for each channel, topographically arranged in bubble plots, for index finger (left) and thumb (right) are displayed. For each subject all conditions (columns, sustained, dynamic, and standard) of γ^*H*^ = 70 − 150*Hz* and the sub-band β_1_ = 12 − 18 Hz or β_2_ = 18 − 24 Hz with the highest significant z-scores are displayed. Bubble size and color is not directly comparable from subject to subject due to different color-bar ranges. For more detailed description see Figure 3.

**Figure 5:**
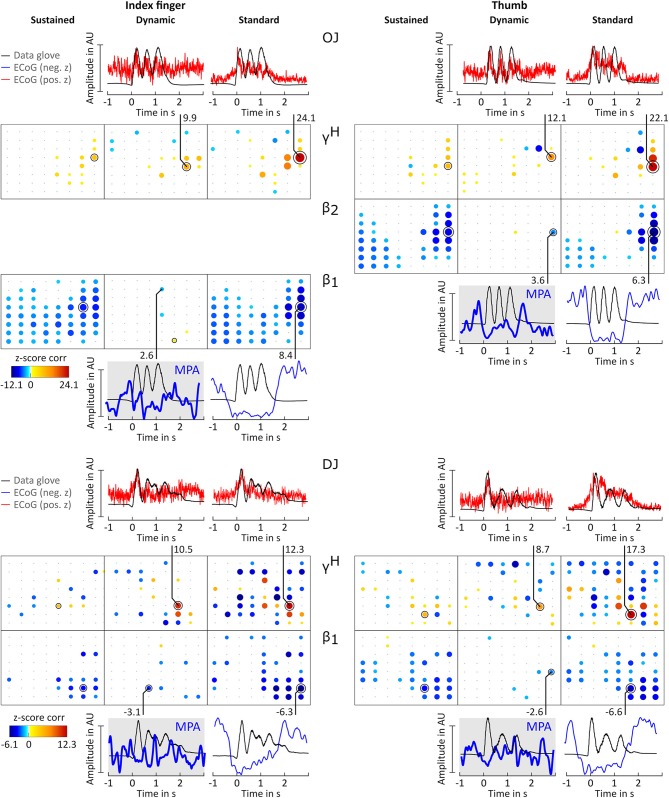
Correlation analysis results and MPA for subjects OJ and DJ. Significant z-score transformed Pearson correlation coefficients for each channel, topographically arranged in bubble plots, for index finger (left) and thumb (right) are displayed. For each subject all conditions (columns, sustained, dynamic, and standard) of γ^*H*^ = 70 − 150*Hz* and the sub-band β_1_ = 12 − 18 Hz or β_2_ = 18 − 24 Hz with the highest significant z-scores are displayed. Bubble size and color is not directly comparable from subject to subject due to different color-bar ranges. For more detailed description see Figure 3.

For all patients and conditions significant negative z-scores were calculated for β_1_, β_2_, β_3_, γ_1_, and γ_2_ sub-bands. High positive z-scores were found in γ^*H*^. $z_{j,ch,fb}^{DYN}$ showed to be much more focal than $z_{j,ch,fb}^{SUS}$ and *z*_*j,ch,fb*_; $z_{j,ch,fb}^{SUS}$ values were comparably lower. Overall z-score magnitude decreases and spatial distribution gets more focal with increasing frequency. The spatial distribution of positive and negative z-scores conforms with the spatial location of ERD and ERS activity. Figure 3B summarizes these findings in detail for patients BP. For each frequency band and condition z-scores are topographically arranged in form of bubble plots. For the remaining subjects only bubble plots for γ^*H*^ and the sub-band with the highest negative z-score over sensorimotor areas are presented (Figures 4, 5). Bubble plots for the remaining frequency bands can be found as Supplementary Information.

Averaged amplitude envelope curves for selected channels and the conditions standard and dynamic are shown in Figures 3B, 4, 5. Channel selection was based on location (only channels located over sensorimotor areas were considered) and maximum absolute z-score magnitude. As reference the averaged original data glove trajectories are visualized. The curves for the standard condition show a sustained decrease during movement. The curves for the dynamic condition show β_1_ MPA and β_2_ MPA. Corresponding z-score values for $z_{j,ch,fb}^{DYN}$ are summarized in Table 1.

**Table 1:** Highest most significant negative z-score for dynamic condition ($z_{j,ch,fb}^{DYN}$) and corresponding frequency band (fb) for each subject and finger movement.

**Subject**	**Index finger**	**Thumb**
	**($z_{j,ch,fb}^{DYN}$, fb)**	**($z_{j,ch,fb}^{DYN}$, fb)**
BP	−2.2, β_1_	−5.3, β_1_
CC	−3.5, β_2_	−3.8, β_2_
ES	−4.9, β_2_	−5.1, β_2_
MN	−2.3, β_1_	−2.3, β_1_
OJ	−2.6, β_1_	−3.6, β_2_
DJ	−3.1, β_1_	−2.6, β_1_

## 4. Discussion

The aim of this study is to investigate whether $\beta^{H}-\gamma_{L}$ MPA, previously observed in EEG (Seeber et al., 2016), are similarly present in ECoG recordings during finger extension and flexion. To tackle this question, ECoG activity and data glove trajectories were split into sustained and dynamic components. The latter was expected to show MPA. Correlations between ECoG and corresponding data glove components were computed. In addition to prominent ERD/ERS phenomena, we indeed found significant correlation between the dynamic ECoG and the dynamic glove data component as shown in Figures 3–5 for index finger and thumb movement, respectively.

ERD/S time-frequency maps (Figure 3A) show the well established patterns of β ERD and high γ ERS (Crone et al., 1998; Pfurtscheller et al., 2003; Scherer et al., 2003; Miller et al., 2007). High gamma activity shows movement related modulation patterns in agreement with previous literature (Schalk et al., 2007; Miller et al., 2009a; Scherer et al., 2009; Hermes et al., 2012).

The position of channels showing significant correlation with behavior were determined based on Talairach coordinates and are located over sensorimotor areas. Using the classical approach of using solely band pass filtered envelope ECoG AMs *Â*_*ch, fb*_, represented by our standard condition, results in higher z-scores compared to sustained ${\hat{A}}_{ch,fb}^{SUS}$ and dynamic ${\hat{A}}_{ch,fb}^{DYN}$ AMs. For the interpretation of the reported z-scores it is relevant to take into account which component (condition) of the data glove signal is compared to which frequency-specific brain feature. For instance, one can find that the high positive correlation for γ^*H*^ standard condition mostly stem from the dynamic movement components. High z-scores in the sub bands standard condition are mainly caused by the rather strong sustained suppression. For the latter high z-scores represent the similarity of the ERD/ERS feature and glove data in general. This distinctions are only possible by decomposing AMs in different components (conditions). There is considerable variability between subjects, but as shown in Figures 3–5 dynamic condition, not only in γ^*H*^, but also in β range sub-bands are modulated by movement. This is in agreement with findings from EEG studies investigating walking (Wagner et al., 2012) and finger tapping (Seeber et al., 2016).

All z-score normalized correlation values reported above were computed at zero-lag between amplitude envelopes and glove data. The impact of time lags on the robustness of the results was analyzed by computing cross-correlation at varying lags. For sustained and standard condition 0s lag showed to result in the highest z-scores whereas for dynamic condition no clear relation could be obtained for all frequency bands. Thus temporal dynamics were analyzed by averaging with 0 s lag. Because of the variability in task execution (high variability in timing and number of finger movements per trial, as can be seen in Figure 2A and in the averaged data glove trajectories ${\bar{\hat{G}}}_{j}$ in Figures 4, 5 for patients CC and DJ) we shall focus our interpretation of results to the period from 0.5 s before start of movement and during the first finger movement cycle. As excepted γ^*H*^ AMs show clear modulation with the movement pace and are thereby highly positively correlated with the finger trajectories. Interestingly for every subject individual electrodes were found with high negative z-values. Corresponding γ^*H*^ AM curves peak directly before movement onset and resemble a rather flat line during the rest of the movement period. An example of an averaged amplitude envelope curve illustrating this phenomena for subject BP thumb movement is presented in Figure 3B. This activity seems to coincide with movement planning. β_1_ AMs in subjects BP, MN and OJ and β_2_ AMs in subjects CC and ES anticipate the motor behavior and are negatively correlated to the first cycle of thumb and index finger movement. These results suggest that β rhythms not only show sustained decrease in amplitude, but that they are superimposed with dynamic modulations that are somewhat correlated with behavior and thus indeed show MPA.

Data analyzed in this paper were originally recorded to study temporal dynamics of γ^*H*^ activity during movement (Scherer et al., 2010) and not to research MPAs. This results in some limitations. Limitations include the short movement duration (~ 2 s), the high variability of motor execution and small number of movement cycles per finger movement (1–6 cycles per trial), the small number of movement trials (23–31) and the short inter-cue interval. We expect that longer trials and larger number of rhythmic finger movements per trial would result in visually much clearer and more consistent dynamic modulations. The use of a general cut-off frequency of 0.4 Hz based on movement speed for low- and high-pass filtering with such a high variability in movement-speed holds another limitation and could further be improved by individualized selection of filter stop/pass bands likely resulting in higher correlation values.

Nonetheless we find and report for the first time dynamic β_1_ and β_2_ modulations that are significantly, but rather loosely, correlated with finger flexion and extension. Yet, their time course and location suggest that they contain information that is different and potentially supplementary to the information that γ^*H*^ modulations provide. Additionally to these novel findings in ECoG, we replicated activity patterns in β and γ^*H*^ that are in agreement to previous literature (Schalk et al., 2007; Miller et al., 2009a; Scherer et al., 2009; Hermes et al., 2012). The model of interpretation of β activity we suggest in this paper may pave the way to gaining a more comprehensive understanding of brain activity in the context of motor behavior. Sound in-depth knowledge of brain activity will lead to more informative BCI features, which represents one essential component toward the improvement of BCI pattern recognition performance in BCI and fBM applications.

## Data Availability Statement

The datasets generated for this study are available on request to the corresponding author.

## Ethics Statement

The studies involving human participants were reviewed and approved by Human Studies Division (Institutional Review Board) of the University of Washington, Seattle, WA, USA. The patients/participants provided their written informed consent to participate in this study.

## Author Contributions

SZ, JO, and RS designed the research. SZ collected the data. JU, MS, and RS analyzed the data. JU, MS, SZ, JO, and RS wrote the manuscript.

### Conflict of Interest

The authors declare that the research was conducted in the absence of any commercial or financial relationships that could be construed as a potential conflict of interest.
