# Supplementary figures and images for: ECoG Beta Suppression and Modulation During Finger Extension and Flexion
Source: Front Neurosci. 2020 Feb 13;14:35. doi: 10.3389/fnins.2020.00035 (PMC7031656; doi:10.3389/fnins.2020.00035)

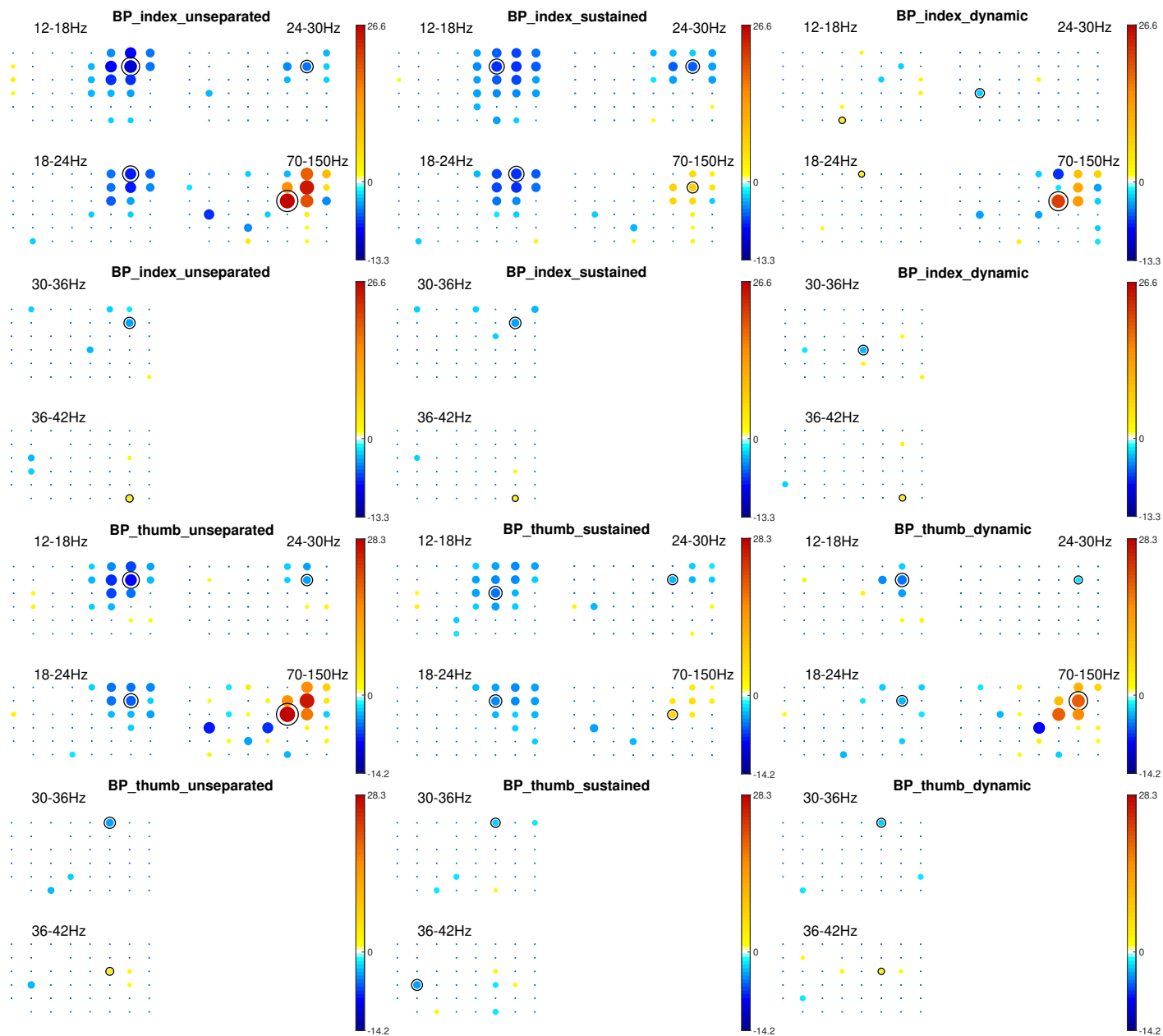

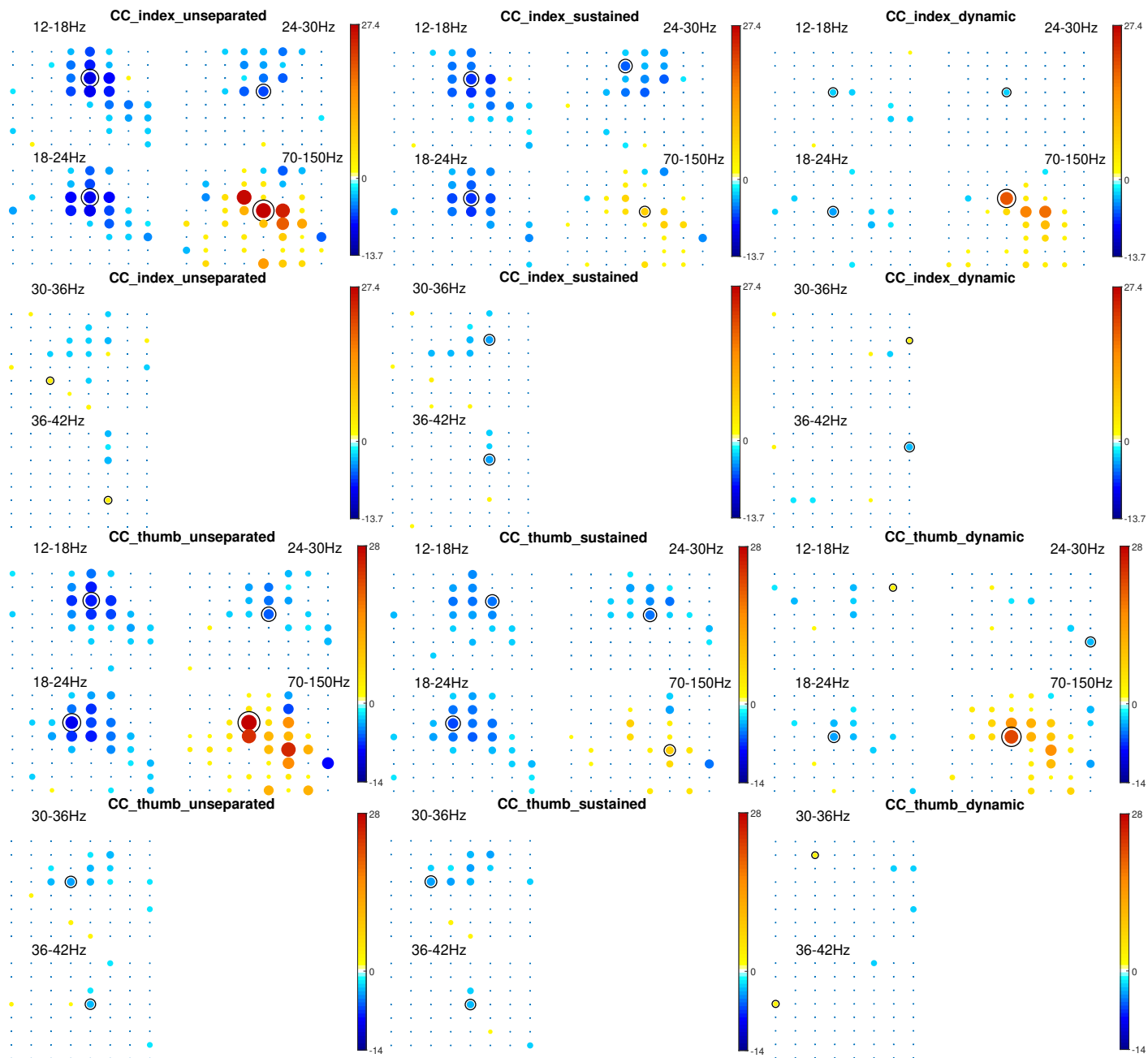

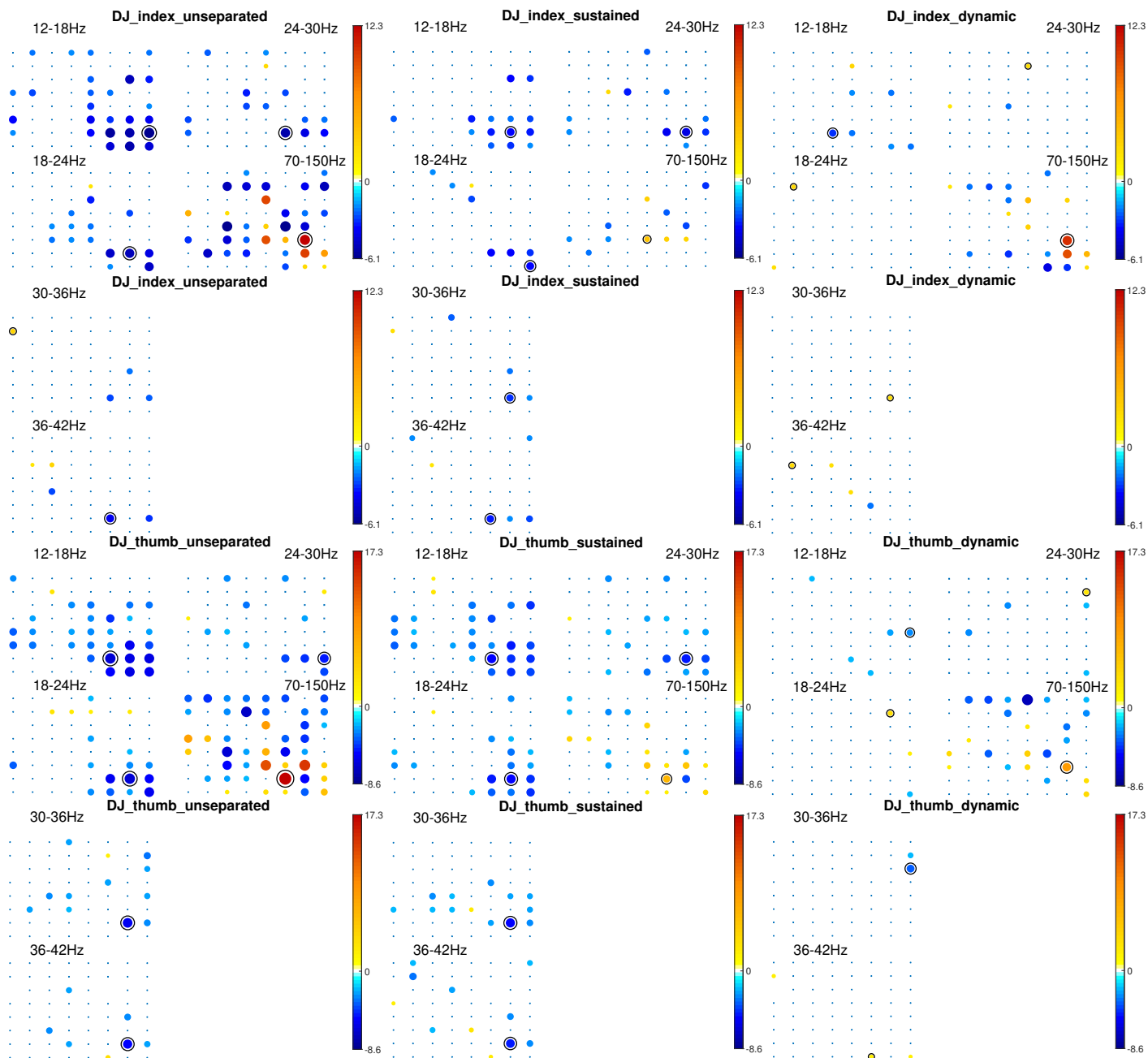

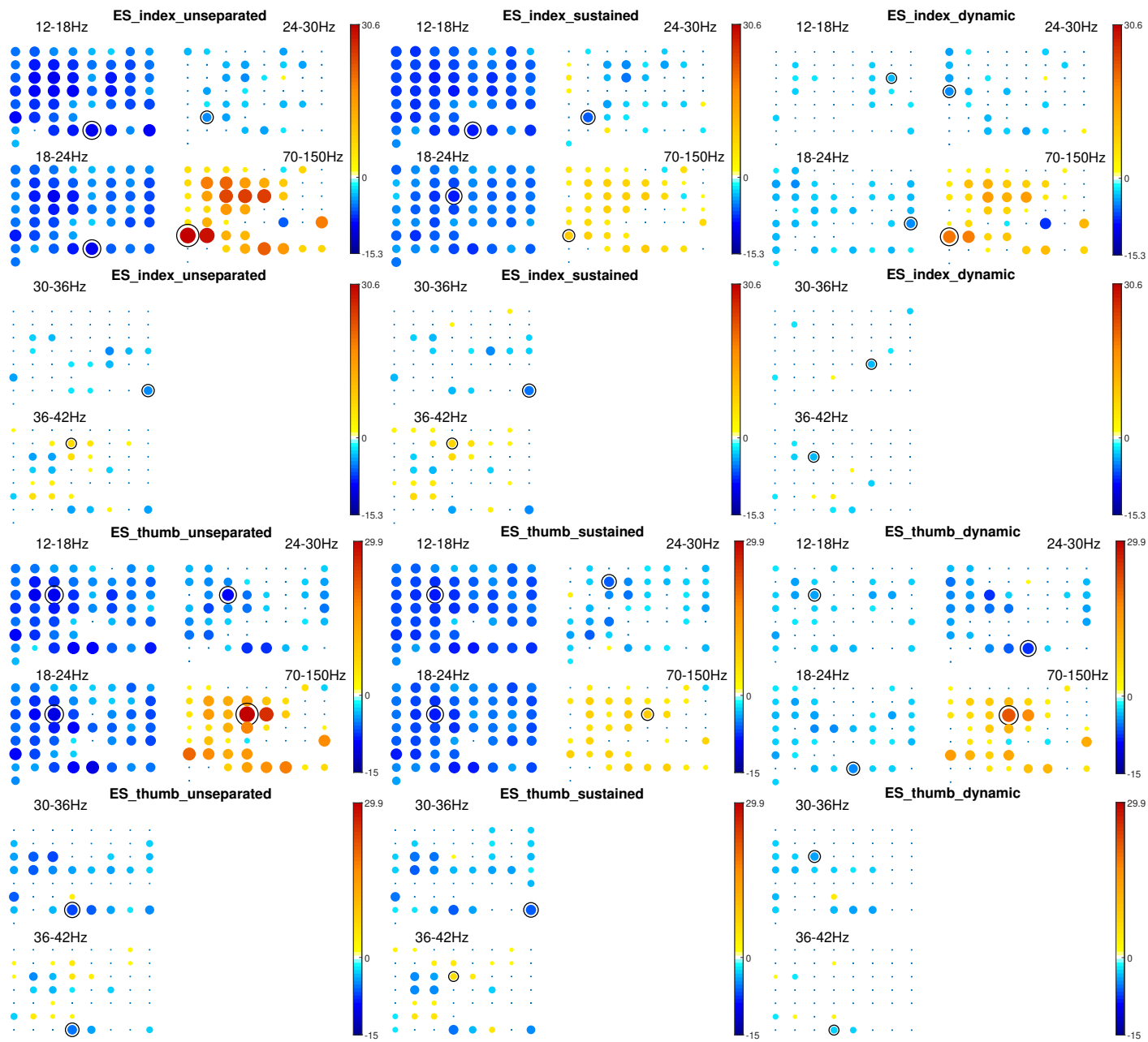

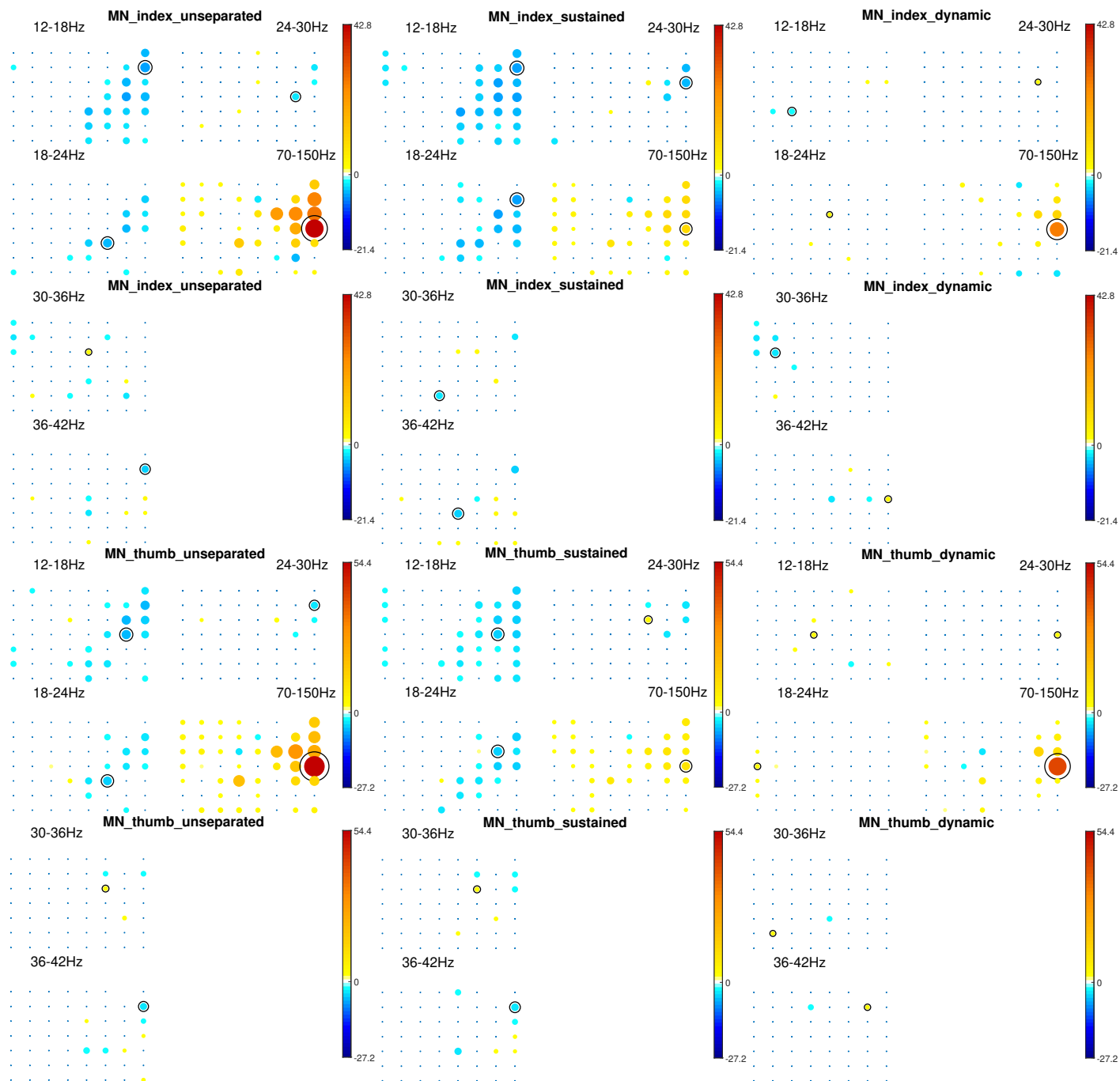

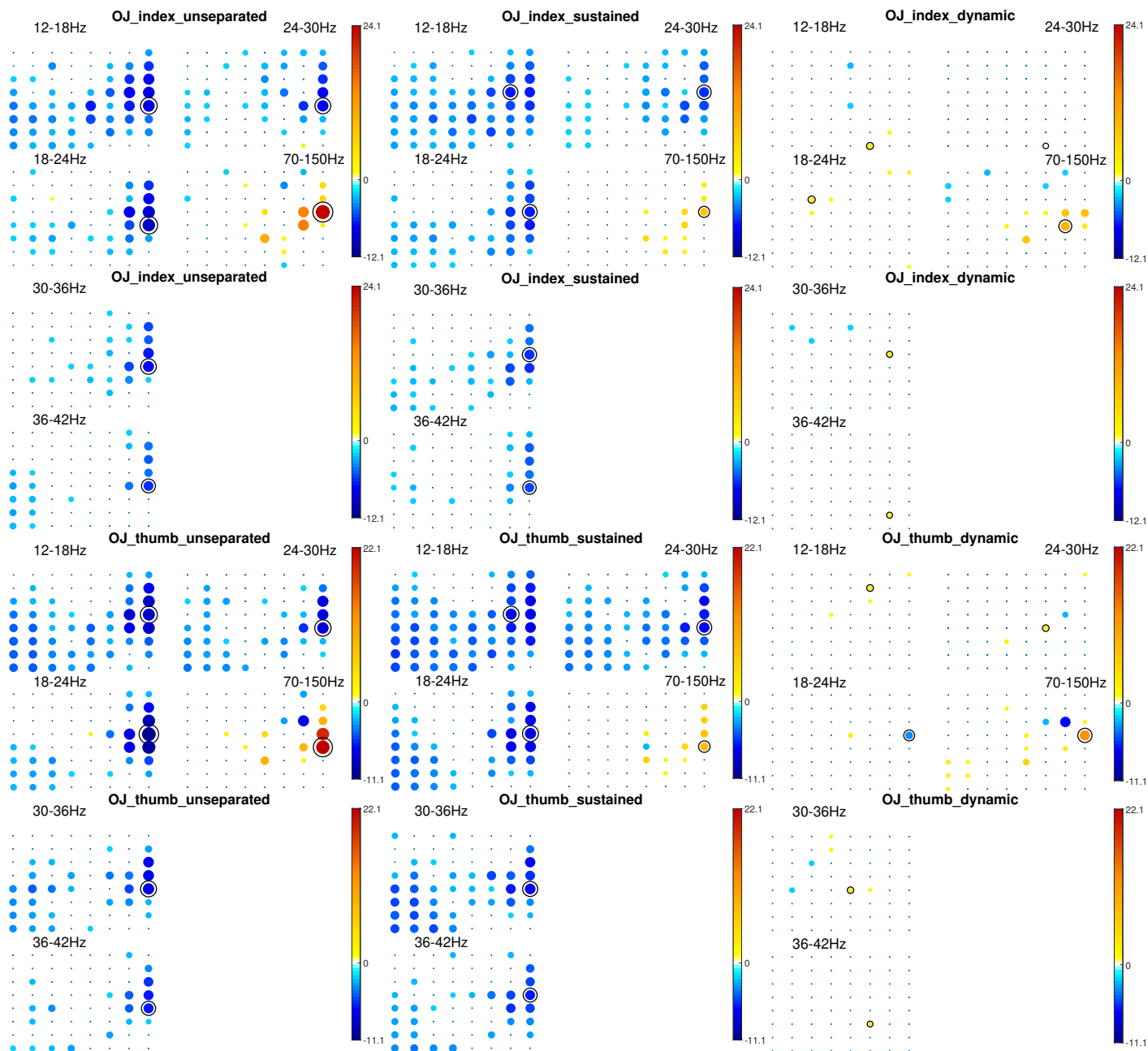

Supplement: Supplementary file 1 [file Data_Sheet_1.PDF]
